# Comparison of efficacy and safety of complementary and alternative therapies for Parkinson's disease: A Bayesian network meta-analysis protocol

**DOI:** 10.1097/MD.0000000000022265

**Published:** 2020-09-18

**Authors:** Chuancheng Li, Hongqiang An, Jiahao Wang, Zhenyuan Jiang, Tianqi Zhang, Qing Huo

**Affiliations:** a First College of Clinical Medicine, Shandong University of Traditional Chinese Medicine, Jinan; c Xintai People's Hospital, Xintai; b Department of Neurology, Affiliated Hospital of Shandong University of Traditional Chinese Medicine, Jinan, Shandong Province, China.

**Keywords:** complementary and alternative therapies, network meta-analysis, parkinson's disease, protocol

## Abstract

**Background:**

In recent years, the incidence of Parkinson's disease (PD) has been on the rise. However, the existing therapy of PD cannot fundamentally treat the disease. Meanwhile, the complementary and alternative therapies of PD have played a positive role in the treatment of PD. Traditional meta-analysis was only able to compare 2 interventions, while the efficacy and safety of many complementary and alternative therapies were not comparable. Therefore, this study compared the efficacy and safety of different complementary and alternative therapies through network meta-analysis (NMA).

**Methods:**

A comprehensive search of randomized controlled trials of complementary and alternative therapies for PD, as well as trials currently in progress, will be conducted until August 2020. Literature and data extraction were independently completed by two researchers. Through the meta-analysis of pairwise comparison and Bayesian NMA, all evidences are comprehensively evaluated. Use STATA15.0 and WinBUGS1.4.3 software for data processing and analysis, and use grading of recommendations assessment development and evaluation to classify the quality of evidence in the NMA.

**Results:**

The aim of this study is to obtain a ranking of efficacy and safety of different complementary and alternative therapies for PD.

**Conclusion:**

Complementary and alternative therapies for PD have positive significance in improving the symptoms of PD, and can provide evidence support for clinicians and patients.

**INPLASY registration number:**

INPLASY202080079

## 1 Introduction

Parkinson disease (PD) is 1 of the most common neurodegenerative diseases in middle-aged and older adults. The pathological manifestation of PD is progressive loss of dopaminergic neurons in the midbrain substantia nigra. The clinical manifestations of PD include motor symptoms and non-motor symptoms. The motor symptoms of PD include static tremors, muscle rigidity, motor retardation, and abnormal posture gait, and non-motor symptoms include depression, sleep disorders, constipation, autonomic nervous dysfunction and so on. PD was the second major type of neurodegenerative disease after Alzheimer's disease.^[1]^ At least 4 million people worldwide are diagnosed with PD,^[2]^ and it is expected to reach 9 million in 2030,^[3]^ and the incidence of PD among people over 65 years of age in China is 1.7%.^[4]^ The pathogenesis and mechanism of PD are still unclear, but the incidence of PD increases with age, and the incidence of PD gradually increases after the age of 50.^[5]^ PD is mostly treated with levodopa preparations, monoamine oxidase inhibitors and other drugs, but they cannot prevent the progression of the disease and have limitations. For example, long-term use of these drugs as the disease progresses will cause motor complications, such as levodopa motor disorders, the appearance of “on” and “off” phenomena,^[6]^ mental illness,^[8]^ and the appearance of constipation, headache, depression, sleep disorders, etc..^[7]^ At the same time, other emerging non-drug treatment methods include microRNA, globus pallidus surgery, thalamotomy, deep brain stimulation, which have become patients’ options, but these methods cannot control the symptoms after drug treatment, and they are still affected by the motor symptoms of PD.^[10]^ The non-motor symptoms of depression and sleep disorders in PD patients are using antidepressants and drugs for the treatment of sleep disorders, which have a side effect on patients.^[9]^ With the gradual deterioration of the physical functions of patients with PD, as well as the economic suffering caused by long-term drug use to the family, the quality of life has declined, and at the same time, it has brought huge pressure to the society. Therefore, more and more patients with PD are seeking methods other than drugs to improve their symptoms.^[10]^

Complementary and alternative therapies play a vital role in the adjuvant treatment of PD, including exercise, acupuncture, moxibustion, massage, Chinese herbal medicine, yoga, Tai Chi, music therapy, etc.^[11]^ Complementary and alternative therapies for PD have played a huge role in delaying the onset of PD and alleviating the disease. Good practices have been obtained in some countries, and they have provided good guidance for the treatment of PD.^[12]^ In Asian countries, the use rates of complementary and alternative therapies in Singapore and South Korea are 61% and 76%, respectively.^[13,14]^ However, in Western countries, the frequency of use is between 34% to 54%,^[15–18]^ and approximately 40% of PD patients in the United States have used at least 1 complementary and alternative therapy for adjuvant treatment of PD.^[18]^ Xiaojiao Xu et al found that proper exercise can alleviate the side effects such as fatigue and movement disorder caused by anti-Parkinson's drugs.^[19]^ LaHue et al conducted a follow-up study on more than 200,000 patients who continuously participated in moderate to severe exercise and found that their risk of PD was 40% lower than that of those who were sedentary and inactive.^[20]^ Acupuncture can have a positive effect on PD, can delay dopamine exhaustion, and can improve the dopaminergic system, enhance neuroprotection, and can improve the gait of patients with PD.^[21]^ Tai Chi has a positive effect on the motor function and balance of patients with PD and can relieve stress, improve the quality of life and mood of patients, and is a safe and feasible way of exercise for patients with mild to moderate PD.^[22]^ Yoga can reduce the risk of falls in patients with PD, and can improve freezing gait and increase the stability of postural balance.^[23,24]^

There are lots of complementary and alternative therapies for PD. Although their efficacy has been evaluated and reported in randomized controlled trials and systematic reviews, it is difficult for many doctors and patients to choose from many methods. Because traditional meta-analysis usually can only compare two interventions, how to screen the most effective and safest method in the face of multiple interventions has become a major clinical problem. The network meta-analysis (NMA) can compare a variety of interventions, and then screen the best and safest interventions. Therefore, this article compares the efficacy and safety of multiple complementary and alternative therapies for PD through the idea of NMA to provide corresponding help for clinicians and PD patients.

## 2 Materials and methods

This study will strictly follow the Preferred Reporting Items for Systematic Review and Meta-Analysis protocols specification for reporting.^[25]^

### 2.1 Study registration

This NMA has been registered on the International Platform of Registered Systematic Review and Meta-analysis Protocols (INPLASY). The registration number is: INPLASY202080079 (URL = https://inplasy.com/inplasy-2020-8-0079/).

### 2.2 Inclusion criteria

#### 2.2.1 Type of study

Complementary and alternative therapies (exercise, acupuncture, moxibustion, massage, Chinese herbal medicine, yoga, Tai Chi, music therapy) for PD all related randomized controlled trials (RCT) and systematic reviews/meta-analysis.

#### 2.2.2 Types of participants

(1) Patients who have been diagnosed with primary PD are classified as grades 1 to 4 on the Hoehn and Yahr scales.^[26]^
(2) Take 1 or more anti-parkinsonian medications regularly.
(3) The age was between 45 and 75.
(4) No restrictions on gender and race.

#### 2.2.3 Interventions and comparisons

The intervention measures in the treatment group include exercise, acupuncture, moxibustion, massage, Chinese herbal medicine, yoga, Taiji, music therapy. All kinds of intervention measures can be used alone or in any combination. However, the treatment group must adopt complementary and alternative therapy for PD based on regular western medicine treatment. The control group only used regular western medicine treatment or selected other methods besides intervention measures on the basis of regular western medicine treatment.

#### 2.2.4 Outcomes

The main results include the total score of the unified Parkinson's disease rating scale scale, the Parkinson's disease questionnaire-39 item -39 score, and the secondary results include the Berg balance scale, the HAMD depression scale score, the PD sleep quality scale score, adverse effects, and other indicators.

### 2.3 Exclusion criteria

Exclude non-RCTs, reviews, animal experiments, and other research, as well as non-English literature and repeated articles or conference abstracts.

### 2.4 Search strategy

The systematic study of literature retrieval skills and precautions before literature retrieval, and formulate the final retrieval strategy after multiple pre-searches. The databases we searched mainly included PubMed, Cochrane Central Register of Controlled Trials, Cochrane Library, EMBASE, Web of Science, and included all RCT of complementary and alternative therapies for PD. The search time is up to July 2020. At the same time, the references included in the systematic review/meta-analysis were tracked. We conduct a comprehensive search by using a combination of medical subject headings (MeSH) and free words, combined with the specifications of related databases. Also, we will also search for ongoing randomized controlled trials, such as trials registered and conducted on the WHO ICTRP (World Health Organization International Clinical Trials Registration Platform). Literature screening was carried out simultaneously and independently by two researchers. If there are disagreements, they can be discussed and resolved, or if necessary, a third researcher should discuss and resolve them and explain the reasons. The specific search strategy of the PubMed database is shown in Table 1.

**Table 1:**
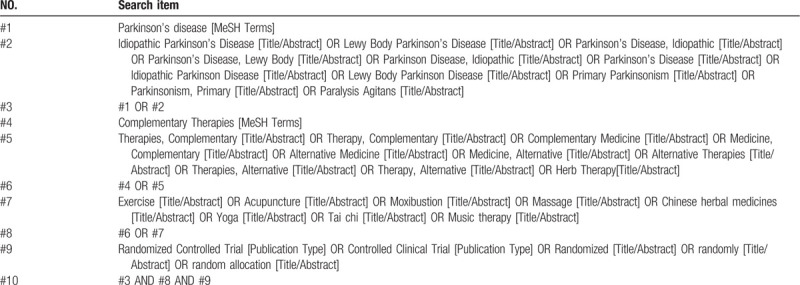
Details of the search strategy for PubMed.

### 2.5 Data extraction

According to the established search strategy, all the researches searched in the above database and manually searched are imported into EndNoteX9 to check and filter the documents and explain the reasons when excluding documents. For all included trials, two researchers independently extracted data through pre-designed data extraction tables and recorded all data using Microsoft Excel 2019 software. The data extracted are as follows:

#### 2.5.1 Publication of information and data extraction information

Title, first author, journal, country, date of data extraction.

#### 2.5.2 Participants

Sample size, race, age, research object source, inclusion criteria, exclusion criteria.

#### 2.5.3 Interventions

Specific intervention details, method of implementation,

#### 2.5.4 Measurement index

Primary and secondary outcomes, odds ratio, mean difference, confidence interval (CI).

If there is a lack of relevant data, we will try to contact the main researcher via email or telephone to obtain the relevant missing information.

### 2.6 Risk of bias assessment

According to the Cochrane Collaboration's bias risk assessment tool, the assessment mainly includes 7 aspects.^[27]^ Each aspect is classified as “Yes”, “No”, and “unclear”. It is conducted independently by two researchers. If they have different opinions, a third researcher will independently review and explain the reasons.

### 2.7 Assessment of heterogeneity

Due to the diversity of our research design, similar studies from different countries or regions are collected together for meta-analysis, there will inevitably be differences. If there is the heterogeneity, we will take the following measures:

#### 2.7.1 Subgroup analysis

When there is heterogeneity between research results, we will conduct a comprehensive and systematic analysis of the reasons for heterogeneity and carry out hierarchical treatment according to different sources of heterogeneity. If it is due to the variation between studies, the intervention measures are consistent, and the research objects come from different populations. The following aspects will be used: age, course of the disease, gender.

#### 2.7.2 Sensitivity analysis

By changing some important factors that may affect the results of the merger, we observe the heterogeneity of different studies and whether the results of the merger have changed, and then judge the stability and strength of the results. For example, for missing data, re-analyze after entering reasonable values, and if there is a heterogeneous change, the missing data will be the source of heterogeneity. If the missing data is re-analyzed with reasonable values, the heterogeneity has not changed, which means that the sensitivity is low and the results are stable and reliable.

### 2.8 Statistical analysis

#### 2.8.1 Statistical model selection

We will select the effect model based on the *I*^2^ value and *P*- value in the heterogeneity test. If *I*^2^ is less than or equal to 50, it means that the heterogeneity is acceptable. When the *P*-value in the heterogeneity test result is less than 0.1, it can be considered that multiple similar studies have homogeneity, and the fixed effects model can be used. When the P-value in the heterogeneity test result is less than or equal to 0.1, the reasons for heterogeneity are firstly analyzed, such as whether the design plan, age, gender, the severity of disease, and other factors are consistent. If the heterogeneity is caused by these reasons, subgroup analysis is used to calculate the combined statistics. If heterogeneity still exists after analysis by these methods, the random effects model is selected.

#### 2.8.2 Pairwise meta-analysis

We will use STATA15.0 software to conduct a pairwise meta-analysis, choose odds ratio value for dichotomous variables as the result expression form, and choose mean difference value as the result expression form for continuous variables. Calculate the 95% CI for each effect indicator. We will use the *I*^2^ value as a measure of the degree of heterogeneity among multiple studies.

#### 2.8.3 NMA

We will use STATA15.0 for NMA, and use a random-effects model to merge data and draw evidence network. The Bayesian NMA is mainly based on the Markov-chain-Monte-Carlo (MCMC), because it is more flexible and can solve the statistical processing in the complex evidence network. At the same time, it can use the posterior probability obtained to rank all intervention measures involved in the comparison and distinguish the good and bad order. Therefore, we will use the MCMC in WinBUGS1.4.3 to perform Bayesian NMA of the random effects model.^[28]^ When running the WinBUGS1.4.3 program, for each MCMC, the number of iterations is run 100,000 times, and the first 5000 times are discarded as the number of annealing. The Brooks-Gelman-Rubin statistical method is used to assess the convergence. At the same time, we will adjust the number of iterations and annealing time according to the specific situation, and calculate the 95% CI of the corresponding effect value. We will use the surface under the cumulative ranking curve values^[29]^ to rank the intervention measures. The surface under the cumulative ranking cure value ranges from 0 to 1. The closer to 1, the better the possibility of intervention becoming the best intervention.

### 2.9 Assessment of inconsistency

When there are closed loops in NMA, we need to assess its consistency.^[30]^ Therefore, we will use the node splitting method to calculate the difference between the direct comparison evidence and the indirect comparison evidence and judge whether there is inconsistency through the *P*-value.

## 3 Assessment of publication bias

In a meta-analysis, a funnel plot is often used to analyze publication bias. The funnel plot is drawn with the effect size of each study as the abscissa and the standard error (SE) of the effect size as the ordinate. Therefore, we will include more than 10 studies to construct a funnel plot. If the funnel plot is symmetrical, it means that the meta-analysis may not have publication bias; if it is asymmetric, there is publication bias, we will analyze the reasons for the publication bias.

## 4 Assessment of the quality of evidence

The grading of recommendations assessment, development, and evaluation (GRADE) method is 1 of the international standards for grading the quality of evidence and the strength of recommendations. It is suitable for systematic reviews, clinical practice guidelines, and health technology assessments.^[31]^ GRADE divides the quality of evidence into 4 grades: high, medium, low, and very low, and the strength of recommendation is divided into 2 grades: strong and weak. Since the NMA is mainly based on RCT, GRADE's evaluation in the NMA mainly includes 5 demotion factors: risk of bias, indirectness, inconsistency, imprecision, and publication bias.^[32]^ GRADE is the most valuable tool for the current NMA to grade the quality of evidence.

## 5 Discussion

PD is 1 of the common degenerative diseases of the central nervous system in middle-aged and elderly people. At present, it is difficult to achieve satisfactory results in drug therapy for PD. The drugs taken by PD will cause various discomforts and even decrease the therapeutic effect with the prolongation of time. In the latest systematic review of complementary and alternative therapies for PD, it has been confirmed that complementary and alternative therapies can play a positive role in improving the symptoms of PD. However, traditional meta-analysis can only compare 2 interventions and cannot compare multiple interventions. So far, there is no NMA to systematically compare various complementary and alternative therapies for PD and evaluate their efficacy and safety. Therefore, this study is the first NMA of complementary and alternative therapies for PD. The purpose of this study is to provide evidence for the efficacy and safety of complementary and alternative therapies for PD and to provide help for clinicians and patients. In this study, although we conducted a comprehensive literature search, and used the Bayesian model to conduct an NMA, and we used SURCA to rank the intervention measures, and use GRADE to evaluate the quality of evidence. But there are inevitably some limitations: For example, first, we only chose English research when choosing the language, which caused publication bias; second, our research is based on literature rather than original data, and there will be some deviation. Complementary and alternative therapies for PD are simple and easy to repeat for most patients with PD, economical, and most patients can achieve it at home. Therefore, through this study, we want to attract the attention of clinicians and patients and provide help for the treatment of PD.

## Acknowledgments

This work was supported by the key R & D program of Shandong Province (2018GSF119021).

## Author contributions

**Conceptualization**: Chuancheng Li, Qing Huo.

**Formal analysis**: Qing Huo, Chuancheng Li, Jiahao Wang.

**Methodology**: Zhenyuan Jiang, Tianqi Zhang.

**Project administration**: Jiahao Wang, Hongqiang An.

**Software**: Hongqiang An, Zhenyuan Jiang, Tianqi Zhang.

**Writing – original draft**: Chuancheng Li.

**Writing – review & editing**: Qing Huo.

## Abbreviations

Abbreviations: CI = confidence interval, GRADE = grading of recommendations assessment development and evaluation, NMA = network meta-analysis, OR = odds ratio, PD = Parkinson's disease, RCT = randomized controlled trials, UPDRS = unified Parkinson's disease rating scale.
